# Cardiovascular Disease Diagnosis from DXA Scan and Retinal Images Using Deep Learning

**DOI:** 10.3390/s22124310

**Published:** 2022-06-07

**Authors:** Hamada R. H. Al-Absi, Mohammad Tariqul Islam, Mahmoud Ahmed Refaee, Muhammad E. H. Chowdhury, Tanvir Alam

**Affiliations:** 1 College of Science and Engineering, Hamad Bin Khalifa University, Doha 34110, Qatar; halabsi@hbku.edu.qa; 2 Computer Science Department, Southern Connecticut State University, New Haven, CT 06515, USA; islamm2@southernct.edu; 3 Geriatric Department, Hamad Medical Corporation, Doha 3050, Qatar; mrefaee@hamad.qa; 4 Department of Electrical Engineering, Qatar University, Doha 2713, Qatar; mchowdhury@qu.edu.qa

**Keywords:** cardiovascular diseases, DXA, retina, deep learning, machine learning, Qatar Biobank (QBB)

## Abstract

Cardiovascular diseases (CVD) are the leading cause of death worldwide. People affected by CVDs may go undiagnosed until the occurrence of a serious heart failure event such as stroke, heart attack, and myocardial infraction. In Qatar, there is a lack of studies focusing on CVD diagnosis based on non-invasive methods such as retinal image or dual-energy X-ray absorptiometry (DXA). In this study, we aimed at diagnosing CVD using a novel approach integrating information from retinal images and DXA data. We considered an adult Qatari cohort of 500 participants from Qatar Biobank (QBB) with an equal number of participants from the CVD and the control groups. We designed a case-control study with a novel multi-modal (combining data from multiple modalities—DXA and retinal images)—to propose a deep learning (DL)-based technique to distinguish the CVD group from the control group. Uni-modal models based on retinal images and DXA data achieved 75.6% and 77.4% accuracy, respectively. The multi-modal model showed an improved accuracy of 78.3% in classifying CVD group and the control group. We used gradient class activation map (GradCAM) to highlight the areas of interest in the retinal images that influenced the decisions of the proposed DL model most. It was observed that the model focused mostly on the centre of the retinal images where signs of CVD such as hemorrhages were present. This indicates that our model can identify and make use of certain prognosis markers for hypertension and ischemic heart disease. From DXA data, we found higher values for bone mineral density, fat content, muscle mass and bone area across majority of the body parts in CVD group compared to the control group indicating better bone health in the Qatari CVD cohort. This seminal method based on DXA scans and retinal images demonstrate major potentials for the early detection of CVD in a fast and relatively non-invasive manner.

## 1. Introduction

Globally, cardiovascular diseases (CVDs) remain the leading cause of mortality and hence increasing healthcare costs in many countries [1]. CVDs have accounted for 32% of the overall global death where two-third occurs in low- and middle-income countries, according to the World Health Organization (WHO) [2]. Furthermore, CVD has caused 38% of the total deaths of people below 70 years old of age (i.e., premature deaths) in non-communicable diseases (NCD) [3]. In the East Mediterranean region, 54% of the total NCD deaths are caused by CVD according to the WHO’s Eastern Mediterranean Regional Office [4]. In the State of Qatar, according to the Planning and Statistics Authority, 33.1% of the total deaths in 2018 were caused by diseases related to the circulatory system including blood pressure, which made CVD the top concern for Qatar in combating NCD [5]. Although diagnosis and treatment methods/tools of CVD have advanced throughout the years, yet many people still go undiagnosed until the occurrence of serious CVD events such as stroke, heart attack, or myocardial infraction [3].

Risk factors such as age, gender, smoking, obesity, diabetes, hypertension, low-density lipoprotein (LDL) cholesterol, and sedentary lifestyle [6] are known to be linked to CVD and some of these factors have been studied and confirmed for the Qatari population [7,8]. In fact, there exists risk score calculators for CVD such as the Framingham Risk Score [9], and ASCVD [10], which use some of the above factors to predict the 10-year risk of developing CVD. There exists multiple clinical biomarkers such as cardiac troponins I and T, C-reactive protein, D-dimer, and B-type natriuretic peptides which are also shown to be linked to CVD [6]. Furthermore, with the advancement of medical imaging techniques, several imaging modalities have been used to diagnose CVD. Imaging modalities such as delayed enhancement cardiac magnetic resonance (DE-CMR) [11], echocardiogram (ECG) [12,13], ultrasound imaging [14], magnetic resonance imaging (MRI) [15,16,17], and computed tomography (CT) [18,19,20], single-photon emission computerized tomography (SPECT) [21,22], and Coronary CT angiography (CCTA) [23] are also used to detect CVDs. Recently retinal fundus image has emerged as a non-invasive data modality to examine the heart condition. Retinal microvascular abnormalities are found to be linked to CVDs [24,25]. For example, [25] showed that generalized arteriolar narrowing, focal arteriolar narrowing, arteriovenous nicking, and retinopathy, which are all abnormalities in retinal vessels, can be used as indicators for cardiovascular diseases. They also showed that Fundus images can capture these abnormalities. In [26], the authors predicted a variety of CVD risk factors such as hypertension, hyperglycemia, and dyslipidemia from retinal fundus images. Moreover, retinal fundus images have been utilized to predict diabetes [27], and CVD risk factors [28,29].

Another imaging technique that could also be used to diagnose CVD events is the dual-energy X-ray absorptiometry (DXA) through lean and fat mass [30] and/or bone mineral density (BMD) [31]. DXA measurements of fat mass have been shown to have a strong correlation with CVD events as well as its risks [32]. Furthermore, a recent study has shown an independent association between lower BMD and high risk of ASCVD events and death among women in South Korea [33]. DXA provides a number of advantages, including the ability to accurately and precisely measure and differentiate fat, lean, and bone components. It can assess the entire body in a single scan, making it fast in the acquisition while less hazardous due to low-radiation. In addition, it is a non-invasive procedure [34].

In this paper, we focused on two imaging techniques, namely DXA scan and retinal fundus images to demonstrate their contribution to diagnose CVD in Qatari population. We selected these techniques considering their non-invasive nature as well as the ease of access to the data source as they have already been incorporated in QBB protocol to collect participants’ health status. Moreover, the capability of the retinal image and DXA scan in CVD diagnosis has not been investigated thoroughly for the Qatari population. The contributions of this work are as follows:

- We proposed a novel technique that uses retinal images to distinguish CVD group from the control group with over 75% accuracy. To the best of our knowledge, this is a seminal work in CVD diagnosis from retinal images.
- We conducted extensive experiments on DXA scan data and showed that it has reasonable discriminating ability to separate CVD group from the control group yielding over 77% accuracy in the Qatari population.
- We proposed a superior multi-modal approach for CVD diagnosis that fuses both tabular (DXA) and image data (retinal images) to distinguish CVD from the control group with an accuracy of 78.3%. Hence, our study proposes a fast and relatively non-invasive approach to diagnose patients with CVD.

## 2. Related Work

### 2.1. Retinal Fundus Images

Retinal fundus images provide an easy-to-use, non-invasive, and accessible way to screen the human eye health [35]. Retinal images allow medical professionals to examine the eye and diagnose diseases such as diabetic retinopathy, hypertension, or arteriosclerosis. They also provide the diameter and tortuosity measurements of the retinal blood vessels enabling the diagnosis of CVD [36]. ML-based techniques have been utilized to process retinal images for risk factor prediction for CVD. Guo et al. [37] presented a study to investigate the association between CVD and series of retinal information, and whether this association is independent of CVD risk factors in patients with type 2 diabetes. The age- and gender-based case-control study recruited 79 eligible patients with CVD and 150 non-CVD (control) and used three stepwise logistic regression models to evaluate CVD risk factors. Area Under the Curve (AUC) was used to evaluate three models having AUC values of 0.692, 0.661, and 0.775, respectively. Results of the first model showed that hypertension, longer diabetes duration and decreased high-density lipoprotein (HDL)-cholesterol were associated with CVD. Furthermore, the second model showed that patients with diabetic retinopathy, smaller arteriolar-to-venular diameter ratio (AVR), and arteriolar junctional exponent (JE) were likely to develop CVD. Finally, the third model showed that CVD is associated with hypertension, longer diabetes duration, higher HbA1c level, smaller AVR, arteriolar branching coefficient (BC) and JE, as well as larger venular length-to-diameter ratio; this shows that retinal information is associated with CVD, however, this association is independent from CVD risk factors in patients having type 2 diabetes, but it needs further investigation. Cheung et al. [29] developed a DL-based model that can measure the retinal vascular calibre from retinal images. The authors used the Singapore I Vessel Assessment (SIVA) to evaluate the assessments given by the DL system with human graders and found a strong correlation between the DL system and human graders. The dataset used in that study was a composite of images from 15 different retinal imaging datasets, totaling over 70,000 images. The results indicated a strong correlation between 0.82 and 0.95 for the system and human utilizing SIVA on various datasets. Furthermore, multivariable linear regression analysis was carried out between CVD risk factors and vessel caliber measurements by both DL system and human. The central retinal artery equivalent (CRAE) and central retinal vein equivalent (CRVE) were both assessed using regression analysis, and the results showed that R-squared ($R^{2}$) for all CVD risk factors was greater while using the DL system compared to human measurement. Zhang et al. [26] published a study that used retinal images to predict hypertension, hyperglycemia, dyslipidemia, and other CVD risk factors. 1222 retinal images were collected from 625 Chinese people. For the creation of the prediction model, the researchers applied transfer learning. For hyperglycemia detection, the model had an accuracy of 78.7% and an AUC of 0.880; for hypertension detection, the model had an accuracy of 68.8% and an AUC of 0.766; and for dyslipidemia detection, the model had an accuracy of 66.7% and an AUC of 0.703. Age, gender, drinking status, smoking status, salty taste, BMI, waist-hip ratio (WHR), and hematocrit were also used to train the model, and it was able to predict them with an AUC over 0.7. Gerrits et al. [38] used retinal images to develop a deep neural network approach for predicting cardiometabolic risk variables. The study employed data from the Qatar Biobank (QBB), which included 3000 people and 12,000 retinal scans. Age, sex, smoking, total lipid, blood pressure, glycaemic state, sex steroid hormones, and bioimpedance measures were all investigated as risk factors. In addition, the study looked into the impact of age and sex as a mediating factor in cardiometabolic risk prediction. When four images were utilized for person level, good results were achieved for predicting age and sex; acceptable results were obtained for the prediction of diastolic blood pressure (DBP), HbA1c, relative fat mass (RFM), testosterone, and smoking habit; nevertheless, poor results were obtained for total lipid prediction. Craenendonck et al. [39] used retinal images to investigate the relationship between mono- and multi-fractal retinal vessels and cardiometabolic risk variables. The study considered data from 2333 QBB participants. To estimate retinal blood vessel topological complexity, mono- and multi-fractal metrics were derived using the dataset in this work. Results from multiple linear regression analysis revealed that there is a significant relationship between one or more fractal metrics and age, sex, systolic blood pressure (SBP), DBP, BMI, insulin, HbA1c, glucose, albumin, and LDL cholesterol. Based on above-mentioned studies, we can observe that there exists multiple studies focusing on CVD risk factor estimation based on retinal fundus images (Table 1). However, to the best of our knowledge, there exists no study that focused on diagnosing CVD by classifying CVD from non-CVD (control) subjects based on retinal images for the Qatari population.

**Table 1:** Summary of DXA and retinal image-based works for CVD associated risk factor; (AUC: Area under the curve, MAE: mean absolute error, N/A: Not available).

Reference	Year	Dataset	Cohort	ML/DL Results	Findings
Retinal Images Data					
[37]	2016	Retina Images	Images of 79 CVD and 150 Non-CVD patients in Hong Kong	AUC: Model 1 (0.692) Model 2 (0.661) Model 3 (0.775)	The paper evaluated three stepwise logistic regression models to investigate CVD association to retinal information and whether the association is independent from having type 2 diabetes. The findings reveal that information obtained from the retina is independently linked to CVD in type 2 diabetic patients.
[29]	2020	Retina Images	>70,000 images (Collection from 15 datasets from multiple countries and ethnicities)	N/A	The paper developed a DL system to assess retinal vessel caliber (which measure microvascular structure changes which are correlated with CVD risk factors. Outcome of the DL system was compared with human graders. Later a comparison with associated risk factors was carried out. In addition, DL system was able to predict CVD risk factors better than or comparable to human graders.
[26]	2020	Retina Images	Retina images of 625 patients from China	Accuracy of 78.7% for hyperglycemia detection, 68.8% for hypertension detection and 66.7% for dyslipidemia detection	The study aimed at predicting hypertension, hyperglycemia, and dyslipidemia, and other CVD risk factors from retinal images. Transfer learning utilized for model development and obtained a good result.
[38]	2020	Retina Images	12,000 images from QBB	AUC (sex): 0.97; MAE (age): 2.78 years; MEA (SBP): 8.96 mmHg; MAE (DBP): 6.84 mmHg; MAE (Hb1Ac): 0.61%; MAE (relative mass): 5.68 units); MAE (testosterone): 3.76 nmol/L	The paper investigated the possibility of fundus images in predicting cardiometabolic risk factors such as age, gender, smoking habit, blood pressure, lipid profile, and bioimpedance using DL
[39]	2020	Retina Images	Images of 2333 participants from QBB	N/A	The study investigated association between cardiometabolic risk factors and mono- and multifractal retinal vessel using retinal images. Fractal metrics were calculated, and then linear regression analysis was carried out. One or more fractals are linked to sex, age, BMI, SBP, DBP, glucose, insulin, HbA1c, albumin, and LDL, according to the findings.
DXA Data					
[40]	2012	DXA Data	409 participants	N/A	The study aims at comparing BMI with direct measure of fat and lean mass to predict CVD and diabetes among buffalo police officers in New York, US. Findings shows a strong correlation of multiple DXA indices of obesity showed with cardiovascular disease.
[41]	2014	DXA Data	616 ambulatory patients who were not pregnant women, or had self-reported cardiac failure, had cardiac-pacemaker or undergone limb amputation.	N/A	The researchers looked at how body composition factors affect BMI and if they may be used as markers for metabolic and cardiovascular health in Switzerland. The researchers observed that fat mass and muscle mass were important nutritional status markers, and they broadened their investigation to look at the impact on health outcomes for all BMI categories. The authors also underlined the need of evaluating body composition during medical examinations to predict metabolic and cardiovascular diseases.
[42]	2016	DXA Data	117 patients with heart failure with preserved ejection fraction	N/A	The study that was conducted on patients from Germany, England and Slovenia aimed to find out how sarcopenia in individuals with heart failure with preserved ejection fraction is related to exercise ability and muscle strength as well as quality of life. It was found that heart failure has a detrimental effect on appendicular skeletal muscle mass,
[43]	2020	DXA Data	570 patients with and without heart failure	N/A	The goal of the study was to see how aging and heart failure treatments affected bone mineral density. Heart failure was observed to be linked to a greater BMD prevalence of osteoporosis. Heart failure exacerbates the loss of mineral bone density that comes with aging.
[44]	2020	Anthropometric & DXA Data	558 participants who were not diagnosed with diabetes, hypertension, dyslipidemia or CVD.	N/A	The study, which was based on Qatari population from QBB, aimed at comparing Anthropometric & DXA Data in predicting cardio-metabolic risk factors. randomly healthy participants were selected. The study revealed a more in-depth relationship between DXA-based assessment of adiposity as a cardio metabolic risk predictor in Qatar compared to anthropometric markers.
[45]	2020	Demographic, Cardio-metabolic and DXA Data	2802 participants from QBB	N/A	The goal of the study was to find the body fat composition cut-off values to predict metabolic risk in the Qatari population. For Qatari adults of various ages and genders, the study developed cut-off values for body fat measurements that may be used as a reference for assessing obesity-related metabolic risks. According to the findings, there is a substantial link between body fatness and the likelihood of developing metabolic illnesses.

### 2.2. Dual-Energy X-ray Absorptiometry (DXA)

Bone densitometry, also known as Dual-energy X-ray Absorptiometry (DXA), is a form of X-ray technology that is used to analyze bone health and bone loss. DXA is currently a widely accepted procedure for measuring BMD [46]. It has been reported that reduced BMD has an adverse effect on cardiovascular system and osteoporosis which poses a risk to patients with heart failure history [47]. Furthermore, another study with 570 patients found that heart failure was linked to a faster loss of BMD, regardless of other osteoporosis risk factors [43].

In another study involving 117 people having the history of heart failure, DXA revealed that heart failure has a negative impact on appendicular skeletal muscle mass [42]. In a study conducted on 409 participants from police officers in buffalo, New York, multiple DXA indices of obesity showed a strong correlation with CVD [40]. Lang et al. [41] discovered that DXA readouts of fat mass and muscle mass were key nutritional status indicators, and they expanded their research to look at the influence on health outcomes for all BMI categories. Furthermore, the authors emphasized the necessity of assessing body composition during medical examinations in order to anticipate metabolic and cardiovascular disorders. It was reported that BMD, muscle mass, and fat content associated with CVD have detrimental impact in patients with advanced CVD stage which can be seen in the above-mentioned research. Reid et al. [48] investigated the use of CNN to predict abdominal aortic calcification (AAC) based on DXA. High AAC values could be used as a predictor of coronary artery calcium, cardiovascular outcome or even death. In their work, they used data of vertebral fracture assessment (VFA) lateral spine images extracted from DXA and an ensemble CNN was used for training and evaluation. Computational prediction showed high correlation with human-level annotation. In Qatar, the use of DXA in the prediction of CVD, its risk factors, and associated medical conditions has just lately gained traction. Bawadi et al. [49] examined data from the QBB in 2019 to investigate if body shape index could be used as a predictor of diabetic mellitus (DM). Kerkadi et al. [44] studied DXA-based measures in cardio-metabolic risk prediction a year later in 2020, and this study revealed a more in-depth relationship between DXA-based assessment of adiposity as a cardio metabolic risk predictor in Qatar. In the same year, Bawadi et al. [45] carried out a study in Qatar on the age and gender-specific cut-off points for body fat among adults. From the above mentioned articles (Table 1) we can say that, DXA data are linked to multiple metabolic risk factors related to CVD. However, to the best of our knowledge, there exists no study that applies machine or deep learning techniques to diagnose CVD using DXA measurements.

### 2.3. Multi-Modal Approaches

Both DXA and retinal image data collection processes are non-invasive and require less preparation overhead; there is no published work that uses a combination of DXA and retinal image data for CVD detection. We, however, present a summary of existing methods (Table 2) that use a multi-modal approach albeit with modalities different from DXA and retinal images. The existing multi-modal based approaches mainly rely on modalities such as Magnetic Resonance Imaging (MRI), X-Ray, Myocardial Perfusion Imaging (MPI), Electrocardiogram (ECG), Echocardiography (ECHO), and Phonocardiogram (PCG) (Table 2). We would like to mention that none of these invasive multi-modal methods are directly comparable to ours due to the use of different modalities than our approach.

**Table 2:** CVD detection based on multi-modal dataset.

Ref.	Year	Country	Fused Data	Results	Summary
[50]	2019	USA	Electronic Health Record (EHR) and Genetic data	AUROC: 0.790 AUPRC 0.285	The study used a 10-year data from HER and genetic data to predict CVD events using random forest, gradient boosting trees, logistic regression, CNN and long short-term memory (LSTM). Chi-squared was used for feature selection on the EMR data. Results show an improved prediction of CVD with AUROC of 0.790 compared to EMR alone (AUC of 0.71) or genetic alone (AUC of 0.698)
[51]	2020	China	Electrocardiogram (ECG), Phonocardiogram (PCG), Holter monitoring, Echocardiography (ECHO), and biomarker levels (BIO)	Accuracy: 96.67 Sensitivity: 96.67 Specificity: 96.67 F1-score: 96.64	The study aimed at the detection of coronary artery disease (CAD). Data from ECG and PCG of 62 patients were used. Furthermore, data were also collected from Holter monitoring, ECHO and BIO. Feature selection was applied to attain optimum features and support vector machine was used for classification. Results show best performance when feature were fused from all sources.
[52]	2020	USA	Sensors (collect blood pressure, oxygen, respiration rate, etc.) and Medical records (history, lab test, etc.)	Results after feature weighting method: Accuracy: 98.5 Recall: 96.4 Precision: 98.2 F1-score: 97.2 RMSE: 0.21 MAE: 0.12	The study aimed at predicting heart diseases (such as heart attack or stroke) using data gathered from sensors and medical records. Features such as age, height, BMI, respiration rate, and blood pressure were extracted, and then data from both sources were fused. Furthermore, conditional probability is utilized for feature weighting to help in accuracy improvement. An ensemble deep learning is then used for the prediction of heart disease.
[53]	2021	Greece	Myocardial Perfusion Imaging (MPI) and Clinical data	Accuracy: 78.44 Sensitivity: 77.36 Specificity: 79.25 F1-score: 75.50 AUC: 79.26	The study aimed at cardiovascular disease diagnosis using MPI and Clinical data. Polar maps were derived from the MPI data and fused with clinical data of 566 patients. Random forest, neural network, and deep learning with Inception V3 were used for classification. Results show a hybrid model of Inception V3 with random forest achieved an accuracy of 78.44% compared to an accuracy of 79.15% achieved by medical experts.
[54]	2021	USA	Electronic medical records (EMR) and Abdominopelvic CT imaging	AUROC: 0.86 AUCPR: 0.70	The study aimed at developing a risk assessment model of ischemic heart disease (IHD) using combined information from patientsí EMR and features extracted from abdominopelvic CT imaging. In this study, CNN used to extract features from images and XGBoost was used as the learning algorithm. Results show an improved prediction performance with AUROC of 0.86 and AUCPR of 0.70
[55]	2021	USA	Genetic, clinical, Demographic, imaging, and lifestyle.	-	The study aimed at evaluating the ability of machine learning in detecting CAD subgroups using multimodal data. The multimodal data consisted of genetic, clinical, demographic, imaging, and lifestyle data. K-means clustering as well as Generalized low rank Modeling were utilized. Results show that 4 subgroups were uniquely identified.

## 3. Materials and Methods

### 3.1. Ethical Approval

This research was carried out under the regulation of Qatar’s Ministry of Public Health. This work was approved by the Institutional Review Board of Qatar Biobank (QBB) in Qatar, and used a de-identified dataset from (QBB).

### 3.2. Data Collection from QBB

The data used in this study was collected from QBB. The details of the data collection protocol adopted by QBB are described in [56,57]. In brief, participants were invited to QBB and they were interviewed by staff nurses to collect their background history. Then multiple lab tests and imaging such as DXA scan and retinal images were collected. The dataset considered for this study is comprised of 500 participants equally divided into CVD group and control group. There were 262 male participants (CVD:Control = 137:125) and 238 female participants (CVD:Control = 113:125) in the studied cohort. Participants were all adult Qatari nationals aged between 18 and 84 years. Overall BMI was higher in CVD compared to the control group (26.10 ± 2.8:23.20 ± 2.8).

### 3.3. DXA Scan Data Preprocessing

The DXA data consisted of measurements related to bone mineral density, lean mass, fat content, and bone area measurements from different body parts of the participants. The dataset was cleaned by removing columns with missing values of more than 50% from each class (i.e., CVD and control). For the remaining columns, the missing values were imputed by the median value. After the data cleaning stage, 122 features were finally selected for this study. Supplementary File S1 provides the summary statistics of these features. The CVD and control groups’ data were then normalized using the min-max normalization technique [58]:

(1) $$x_{i}^{{}^{\prime }}=\frac{x_{i}-min_{i}\left\{x_{i}\right\}}{max_{i}\left\{x_{i}\right\}-min_{i}\left\{x_{i}\right\}}$$

where *x* denotes the value of a specific DXA feature and max{x} and min{x} denote the largest and smallest values of that feature, respectively.

### 3.4. Retinal Image Collection and Preprocessing

**Figure 1:**
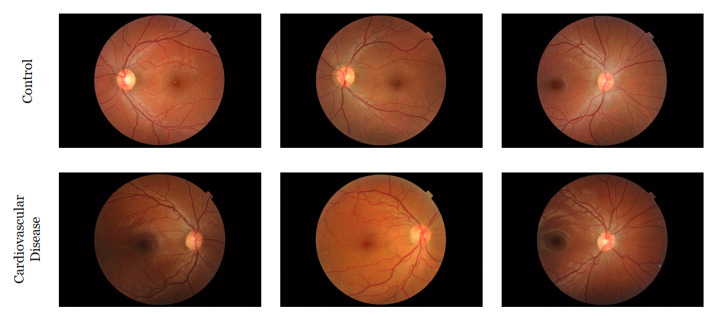
Some randomly selected images from the QBB retinal image dataset.

The retinal fundus images were acquired at QBB utilizing a Topcon TRC-NW6S retinal camera to capture the “microscopic" characteristics of the optic nerve and macula of the participants. At least two images (one for each retina) were collected from each participant, but in some cases, multiple images (three or four) were collected from both eyes. Both (a) macula-centered images and (b) disc-centered images were captured for both eyes. A few participants had no retinal images and we discarded them from our analysis. Figure 1 shows few randomly selected images from each group.

**Figure 2:**
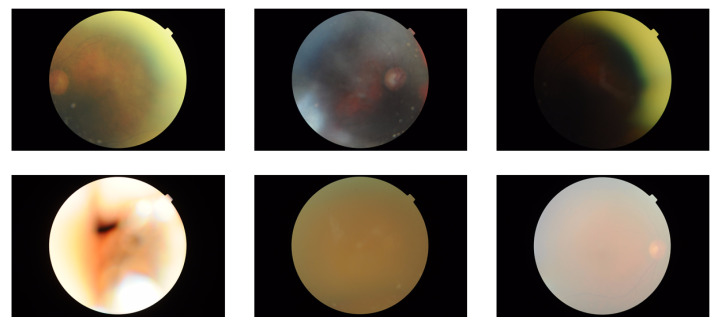
Examples of few low-quality images.

In the dataset, we had 1839 retinal images from all participants. Then, we removed low quality images by visual inspection since they may have negative impact on the downstream classification task. After removing low quality images (all were from the CVD group only), the number of images was reduced to 1805 (874 and 931 images from CVD and control group, respectively). For some participants all their images were removed due to low quality, and therefore, the number of participants in our study was reduced to 483, where 250 were from control and 233 were from CVD group. Examples of some low-quality images can be seen in Figure 2.

## 4. Experiment Setup

To investigate the effect of different types of input data on the outcome of our study, we conducted multiple experiments, both uni-modal and multi-modal. Specifically, we used the tabular and image datasets in isolation in the uni-modal experiments and a combination of them in the multi-modal one. This resulted in three experiment configurations: (i) DXA model: applying traditional machine learning techniques on the DXA data. (ii) Retinal image model: applying deep learning on the retinal images. (iii) Hybrid model: applying deep learning on both the tabular DXA data and the retinal image data. All experiments were performed on an Intel(R) Core(TM) i9 CPU @ 3.60 GHz machine with 64 GB RAM and equipped with NVIDIA GeForce RTX 2080 Ti GPU. For implementation, We used Scikit-learn for the DXA model and fastai for the other two models. Details about each experiment is given below in the following sub-sections.

### 4.1. DXA Model

In the first experiment, we applied six different machine learning algorithms: Decision Tree (DT) [59], (Shallow) Artificial Neural Network (ANN) [60], Random Forest (RF) [61], Extreme Gradient Boosting (XGBoost) [62], CatBoost [63], and Logistic Regression (LR) [64]. We utilized the GridSearchCV utility from Python’s Scikit-learn package for hyperparameter tuning with nested cross validation [65]. Supplementary File S2 includes all the tuned parameters for the ML models.

### 4.2. Retinal Image Model

**Figure 3:**
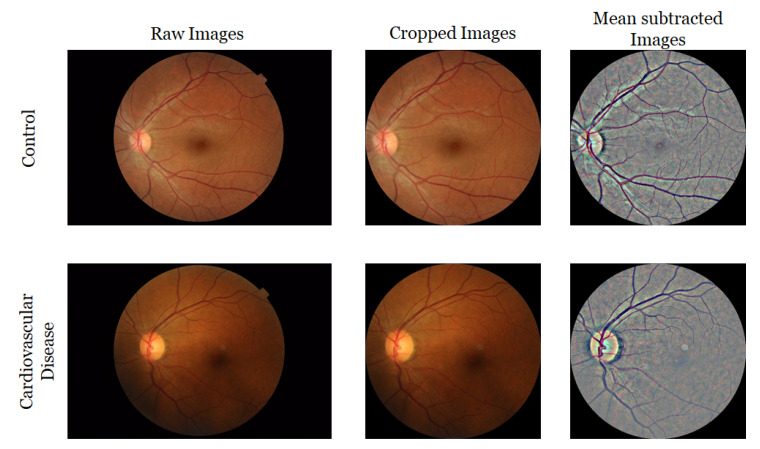
Example of images from QBB dataset before and after pre-processing.

In the second experiment, we applied DL-based techniques to distinguish CVD group from the control group based on the retinal images only. For this experiment, two different image pre-processing steps were applied to generate two sets of images. For the first set, the circular region of each image was extracted. Then, we removed the border-noise by cropping the outside 10% of each image. For the second set, for each cropped image, local mean was subtracted from a 4 × 4-pixel neighborhood, and then placed on a dark background within a square-shaped image with tight boundaries. At the end, we had all images with a size of 540 × 540 with black background. Figure 3 presents samples of original images and pre-processed images. We also applied data augmentation techniques such as random horizontal flip as well as a random brightness and contrast perturbation to enhance the robustness of the model. We experimented with eight popular image classification models, namely, AlexNet [66], VGGNet-11 [67], VGGNet-16 [67], ResNet-18 [68], ResNet-34 [68], DenseNet-121 [69], SqueezeNet-0 and SqueezeNet-1 [70]. We used super-convergence in our experiments which allowed the network to converge faster [71]. The model was fine-tuned through 10 epochs, with all layers (except the last layer) frozen using a one-cycle policy [72] for scheduling the learning rate with a maximum learning rate of 0.01 and a batch size of 8. The model took around 30 min to train.

### 4.3. Hybrid Model

**Figure 4:**
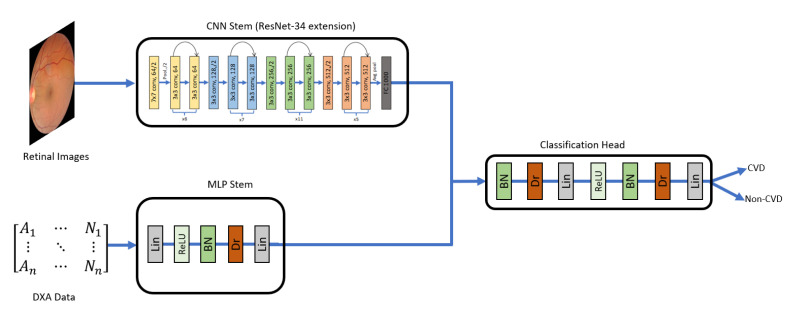
Hybrid model to distinguish CVD from non-CVD using Retinal Images and DXA tabular data. CNN stem for Retinal Image was based on ResNet-34 architecture [68]. The MLP stem includes Linear (Lin), Batch Normalization (BN), Dropout (Dr) layers as shown in the diagram. Both stems were integrated, and their output was fed into the Classification Head having multiple layers of BN, Dr, Lin, ReLU, BN, Dr, and finally a single linear layer as output layer (CVD or non-CVD).

In the third experiment setup, we aimed to combine the DXA and the retinal image data in a deep learning-based approach. To this end, we designed a deep neural network that accepted a multi-modal input. The network consisted of three components: the CNN stem, the MLP stem, and the Classification Head (Figure 4). The CNN stem was responsible for processing the retinal images, while the MLP stem processed the DXA data. Hence, in our work, the fusion between the two data modalities does not take place at image-level. The data from the two different modalities, tabular data (DXA) and images (retinal fundus images) are passed through the two stem networks (MLP stem and CNN stem, respectively) and feature vectors produced from these are fused (vector concatenation) to form a single feature vector which is finally passed through the Classification Head to produce the output classification probability. The CNN stems we experimented with extensions of AlexNet, VGGNet-11, VGGNet-16, ResNet-18, ResNet-34, DenseNet-121, SqueezeNet-0, and SqueezeNet-1. We tried with multipl configurations of MLP stem and Classification Head with different number of layers and neurons. The best configuration we found for MLP stem, and the Classification Head is shown in Table 3.

For the retinal images, we used the cropped images and the mean subtracted images shown in Figure 3 and concatenated them with the tabular data. To achieve this, we used fastai and the image_tabluar library (https://github.com/naity/image_tabular, accessed on 15 August 2021) to integrate the image data and the DXA (tabular) data. Figure 4 shows a high-level diagram of our proposed network architecture for the hybrid model. For the CNN stem, multiple data augmentation techniques such as brightness, contrast, flipping, rotation, and scaling of the images were applied.

The hybrid model was fine-tuned through 10 epochs, with all layers (except the last layer) frozen using a one-cycle scheduler and a 1 × 10${}^{-2}$ learning rate and a batch size of 64. Then, after unfreezing the whole network, 10 epochs were employed for training utilizing discriminative learning rates in the range of (1 × 10${}^{-3}$ and 1 × 10${}^{-2}$). With the 20 epochs, the model took around 30 min to train.

### 4.4. Performance Evaluation Metrics

We evaluated the models with 5-fold cross validation (CV) [65] for all models. The models were evaluated on accuracy, sensitivity, precision, F1-score, and Matthews Correlation Coefficient (MCC). These metrics are highlighted in the following equations:

(2) $$\mathrm{Accuracy}=\frac{tp+tn}{tp+tn+fp+fn}$$

(3) $$\mathrm{Sensitivity}(\mathrm{recall})=\frac{tp}{tp+fn}$$

(4) $$\mathrm{Precision}=\frac{tp}{tp+fp}$$

(5) $$F1\text{-}\mathrm{score}=\frac{2\times \mathrm{Precision}\times \mathrm{Recall}}{\mathrm{Precision}+\mathrm{Recall}}$$

(6) $$\mathrm{MCC}=\frac{\left(tp\times tn)-(fp\times fn\right)}{\sqrt{\left(tp+fp)(tp+fn)(tn+fp)(tn+fn\right)}}$$

where true positive, false negative, false positive, and true negative are represented as *tp*, *fn*, *fp*, and *tn*, respectively. Moreover, we computed an empirical *p*-value for evaluating the significance of a cross-validated performance scores with permutations

## 5. Results

### 5.1. Experimental Results from the Machine Learning Models

The results obtained from the three experiments are presented in this section. For the DXA Model, an ablation study was conducted on different feature types of DXA separately and on all of them with 5-fold CV. Results based on the ablation study shows that the area measurements features have low contribution with 66.5% accuracy. body fat composition, BMD, and lean mass achieved highest accuracy of 77.0%, 73.2%, and 70.2%, respectively. Considering all features (122 features), XGBoost model performed the best achieving highest accuracy of 77.4%. The highest F1-score of 76.8% and the highest MCC of 55.5% (see Table 4). For all DXA based experiments, a *p*-value of < 0.05 was obtained indicating their statistical significance.

For the Retinal Image Model, where retinal images were used only to distinguish CVD form control group, DenseNet-121 model achieved the highest accuracy of 75.6% on the cropped image set based on 5 fold CV. DenseNet-121 model achieved 73.0% accuracy on the mean subtracted image set. Table 5 highlights the comparison of performance among all DL models used in this study. For all retinal image-based experiments, a *p*-value of <0.05 was obtained indicating their statistical significance.

Finally, for the Hybrid Model, where both DXA tabular data and retinal images were combined, ResNet-34 achieved the highest accuracy of 78.3% with 5-fold CV. Table 6 shows a comparison between the eight DL models that we tested in this experiment. For all hybrid model based experiments, a *p*-value of <0.05 was obtained indicating their statistical significance. We also calculated the area under of curve (AUC) of receiver operating characteristics (ROC) for all DL models in this experiment for the cropped images (Supplementary File S3).

### 5.2. Performance of the Hybrid Model Based on Gender and Age Stratified Dataset

**Figure 5:**
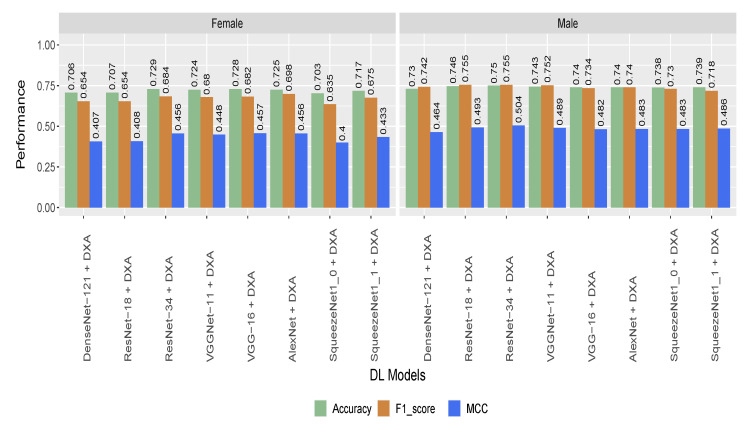
Performance of the hybrid models on gender-stratified participants (based on cropped image).

To check the effectiveness of the proposed model developed on different subgroups of population, we tested the Hybrid Model (incorporating the DXA and retinal image) on age- and gender-stratified samples. Though the performance of the model dropped slightly on the gender-stratified dataset, ResNet-34 based model achieved the highest performance with 75% and 72.9% accuracy for male and female, respectively (Figure 5). For the gender-stratified samples, all the eight models achieved better performance for males compared to females (Figure 5).

**Figure 6:**
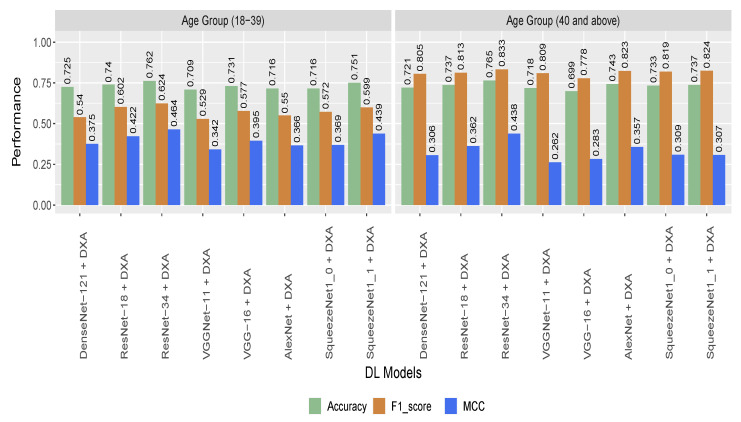
Performance of the hybrid models on age-stratified participants (based on cropped image).

Table 7 provides information on the numbers of participants and images used for different age groups. For the age-stratified samples, the performance of the model was close to the performance of the model while whole dataset. The highest performance across all DL models were obtained from ResNet-34 model with 76.5% accuracy for the 40 and above age group. For the other age group (below 40), the ResNet-34 model also achieve best performance with 76.2% accuracy. Figure 6 shows the detailed results and comparison of the model performance for age-stratified participants.

### 5.3. Statistical Analysis on DXA Data

We conducted statistical analysis on the DXA data for comparing the CVD group against the control group. After analyzing the 122 features, 95 features were statistically significant (*p*-value < 0.05) (Supplementary File S1).

When compared against the control group, CVD group accumulated more fat in different body parts including the arms, legs, trunk, android, gynoid, and android visceral.

Measurements of lean mass in android, leg, arm, trunk, etc., were also higher in the CVD group. For instance, lean mass in trunk (20,329.32 ± 3809:18,779.82 ± 3965) was greater in CVD than the control group. Overall, the total fat mass (25,908.58 ± 6342:20,962.12 ± 4946) as well as total lean mass (43,643.57 ± 8617:40,614.88 ± 9059) was higher in the CVD group and higher BMI value in CVD group reflect their fat and lean mass content.

We also observed higher level of BMD and anthropometric measurements from different body parts in the CVD group compared to the control group (Supplementary File S1). For BMD measurements in different body parts, e.g., head, arms, spine, troch, and trunk, the CVD group had a greater BMD than the control group. Overall the total BMD (1.20 ± 0.11:1.17 ± 0.12) was higher in the CVD group compared to the control group. Individuals with higher weight and BMI tend to have high BMD which might help to reduce the risk of bone fracture [73,74,75]. A similar trend was seen in our cohort.

Furthermore, when it comes to anthropometic measurements, we observed larger bone area in the troch, lumbar spine (L1, L2, L3, L4), and pelvis body parts for CVD group compared to the control group. In summary, most measurements for BMD, fat content, muscle mass, and bone area were higher in the CVD group compared to the control group, according to the analysis. Details of the analysis can be found in Supplementary File S1.

### 5.4. Class Activation Map for Highlighting the Region of Interest in CVD Patients

**Figure 7:**
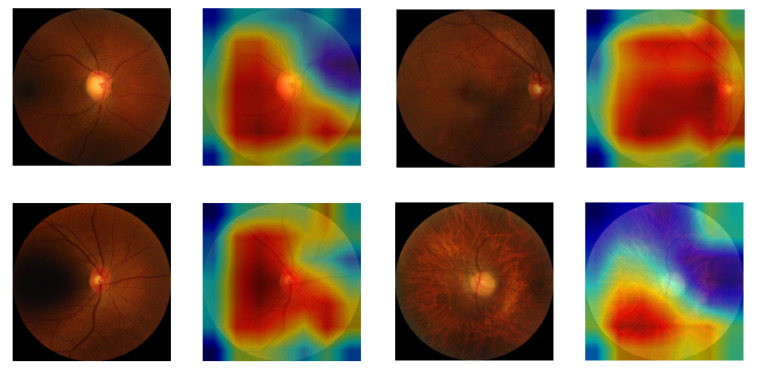
Few retinal images from CVD group with overlaid GradCAM. Red-ish color indicates higher influence on the decision of prediction model compared to the blue-ish color that indicate less influence.

We used Gradient-weighted Class Activation Mapping (GradCAM) [76] to highlight regions of interest which influenced the DL model to make predictions. Figure 7 shows results of GradCAM on images of the CVD class. It was observed that regions of interest, as shown in the color-coded heat map on the right of each image, are mostly in the central region of the retina. Micro hemorrhage can be observed in all images and especially in the bottom left images which is associated with hypertension [77,78].

## 6. Discussion

### 6.1. Principal Findings

Integrating retinal images with DXA improves the performance of CVD detection. In our experiments, the classification accuracy for the retinal image data only and for the DXA data only was 75.6% and 77.4% respectively. However, when both datasets were integrated using a joined deep learning model, there was an improvement in the performance which has reached up to 78.3% accuracy with ResNet-34 based model. This indicates that integration of multi-modal dataset has improved the performance of the proposed model. For the gender-stratified participants, the proposed model achieved almost similar performance for both genders (Figure 5). The performance of the model based on the age-stratified participants were close to the performance of the model with all participants (Figure 6). This indicates that the performance of the proposed model is unbiased towards age-stratified adult population.

The majority of DXA readings in the CVD group were higher than the control group. For statistically significant variables, majority of the BMD, fat content, muscle mass, and bone area measurements for the CVD group had greater average values than the control group (Supplementary File S1). Obesity was shown to be linked as a protective factor of osteoporosis through different mechanisms including mechanical and biochemical mechanisms [79]. In the current study, although obesity (higher BMI) has deleterious effect on cardiovascular risk factors, we can also observe its protective effects on bone health. In summary, our results indicate a better bone health condition for the CVD group which had higher BMI than the control group. Ablation study revealed the better discriminatory power of fat content and BMD than muscle mass and bone areas (Table 4). This highlights the discriminatory power of DXA measurements which could open new avenues in the diagnosis plan of CVD.

### 6.2. Comparison against Other Tools

We could not find any work that used retinal images and DXA for CVD detection based on the Qatari population or outside Qatar. Published work that has been reviewed focused on the prediction of risk factors associated with CVD (refer to Table 1) instead of predicting CVD directly, and therefore, we could not compare the performance of the proposed model against any existing model.

We, however, present a summary of the existing methods (Table 2) that use a multi-modal approach albeit with modalities different from DXA and retinal images. Therefore, we would like to re-iterate that a direct comparison of our proposed multi-modal approach with any of these would not be fair due to the following reasons: First, none of the existing research uses the particular combination of multi-modal data (DXA and retinal images) in their work. To the best of our knowledge, ours is the first research that considers these two data modalities using a machine learning approach. Second, the data used in the approaches (Table 2) include bio-markers related directly to the health of the heart, and hence have an unfair advantage in CVD prediction over our approach at the cost of being more invasive. Our method stands superior in scenarios involving health centers located at remote regions with access to limited non-invasive resources.

### 6.3. Motivation for Using a Multi-Modal Approach

We were motivated to use a “multi-modal” approach to predict CVD using retinal images and DXA data for multiple reasons. First, we have shown that a multi-modal approach is a superior technique (Table 6) than the uni-modal counterparts (Table 4 and Table 5) for predicting CVD. Second, the particular combination of the modalities (DXA and retinal images) we used in our work has never been explored, hence providing a completely novel insight into the possible ways of predicting CVD in a non-invasive manner. Last but not the least, since neither DXA nor retinal data collection is invasive, our proposed multi-modal approach is also, overall, non-invasive, and hence is applicable to a wide variety of health centers possibly located in remote regions with limited resources.

## 7. Limitations

In this work, we used dataset from QBB that consisted of participants from Qatar only. This means that the outcome of this study could be specific to the Qatari population and people of gulf countries with similar lifestyle and ethnicity. This indicates that the results from this study might not be generalized to other population. Moreover, the human retinal images contain a vascular tree that orchestrate a complex branching pattern. To identify different aspects of this tree it is important to have retinal images with high contrast, color balance, and quality. Hence, having more and better quality controlled images could have pushed the accuracy of the proposed model even higher. Despite these limitations, this study serves as a proof of concept that incorporating information from retinal images and DXA provides a novel avenue to diagnose CVD with a reasonable accuracy in a non-invasive manner.

## 8. Conclusions

In this work, we presented a novel deep learning based model to distinguish CVD group from control group by integrating information from retinal images and DXA scans. The proposed multi-modal approach achieved an accuracy of 78.3% which performed better than the individual uni-modal (DXA and retinal images) models. To the best of our knowledge, this is the first study to diagnose CVD from DXA data and retinal images. The achieved performance demonstrated that signals from fast and relatively non-invasive techniques such as DXA and retinal images can be used to diagnose patients with CVD. The findings from our study are required to be validated in a clinical setup for proper understanding of their links to CVD diagnosis. 

## Figures and Tables

**Table 3:** Details of the layers in the MLP stem and the Classification Head of Hybrid model.

Layer Name		Output Size
MLP Stem		
Linear		8
ReLU		8
BatchNorm1d		8
Dropout		8
Linear		8
Classification Head		
BatchNorm1d		264
Dropout		264
Linear		32
ReLU		32
BatchNorm1d		32
Dropout		32
Linear		2

**Table 4:** Performance of ML techniques for ablation study on DXA Model.

Property (No of Features)	Evaluation Metric	DT	MLP	RF	LR	CatBoost	XGBoost
Bone Mineral Density (55)	Accuracy	0.620	0.682	0.686	0.732	0.710	0.726
	Sensitivity	0.617	0.574	0.647	0.678	0.635	0.672
	Specificity	0.628	0.795	0.724	0.785	0.784	0.780
	Precision	0.624	0.740	0.701	0.758	0.746	0.758
	F1-score	0.609	0.639	0.672	0.716	0.685	0.710
	MCC	0.250	0.382	0.373	0.466	0.424	0.456
	*p*-value	1.996 × 10${}^{-3}$	1.089 × 10${}^{-2}$	1.664 × 10${}^{-3}$	1.536 × 10${}^{-3}$	6.623 × 10${}^{-3}$	1.332 × 10${}^{-3}$
Body Fat Composition (15)	Accuracy	0.720	0.770	0.746	0.742	0.740	0.754
	Sensitivity	0.594	0.640	0.697	0.741	0.673	0.723
	Specificity	0.832	0.902	0.789	0.749	0.806	0.787
	Precision	0.790	0.867	0.770	0.747	0.777	0.767
	F1-score	0.669	0.734	0.731	0.741	0.720	0.743
	MCC	0.445	0.560	0.489	0.488	0.482	0.508
	*p*-value	1.248 × 10${}^{-3}$	1.175 × 10${}^{-3}$	1.110 × 10${}^{-3}$	1.052 × 10${}^{-3}$	4.975 × 10${}^{-3}$	9.515 × 10${}^{-4}$
Lean Mass (7)	Accuracy	0.576	0.652	0.690	0.634	0.668	0.702
	Sensitivity	0.556	0.652	0.674	0.613	0.702	0.669
	Specificity	0.596	0.664	0.710	0.657	0.638	0.736
	Precision	0.582	0.717	0.699	0.641	0.661	0.716
	F1-score	0.560	0.650	0.683	0.625	0.678	0.691
	MCC	0.156	0.329	0.386	0.270	0.342	0.406
	*p*-value	9.083 × 10${}^{-4}$	6.950 × 10${}^{-3}$	8.326 × 10${}^{-4}$	7.994 × 10${}^{-4}$	3.984 × 10${}^{-3}$	7.402 × 10${}^{-4}$
Area Measurements (45)	Accuracy	0.580	0.614	0.600	0.664	0.644	0.598
	Sensitivity	0.546	0.528	0.624	0.665	0.660	0.605
	Specificity	0.623	0.698	0.584	0.667	0.633	0.597
	Precision	0.597	0.636	0.602	0.675	0.644	0.598
	F1-score	0.556	0.575	0.607	0.664	0.647	0.598
	MCC	0.175	0.230	0.210	0.335	0.295	0.203
	*p*-value	7.138 × 10${}^{-4}$	4.135 × 10${}^{-3}$	6.662 × 10${}^{-4}$	6.447 × 10${}^{-4}$	3.332 × 10${}^{-3}$	6.057 × 10${}^{-4}$
All (122)	Accuracy	0.672	0.750	0.748	0.768	0.750	0.774
	Sensitivity	0.658	0.656	0.704	0.761	0.694	0.754
	Specificity	0.691	0.855	0.797	0.780	0.812	0.800
	Precision	0.694	0.827	0.777	0.777	0.789	0.790
	F1-score	0.663	0.722	0.736	0.766	0.734	0.768
	MCC	0.363	0.526	0.504	0.542	0.511	0.555
	*p*-value	5.879 × 10${}^{-4}$	5.711 × 10${}^{-4}$	5.552 × 10${}^{-4}$	5.402 × 10${}^{-4}$	2.849 × 10${}^{-3}$	5.126 × 10${}^{-4}$

**Table 5:** A Comparison on the performance of deep learning models built based on retinal images.

Type of Images	DL Model	Accuracy	Sensitivity	Specificity	Precision	f1 Score	MCC	*p*-Value
Cropped images	DenseNet-121	0.756	0.753	0.758	0.74	0.746	0.511	2.023 × 10${}^{-10}$
	Resnet-18	0.694	0.735	0.656	0.661	0.696	0.392	1.732 × 10${}^{-2}$
	ResNet-34	0.753	0.682	0.817	0.773	0.725	0.505	8.824 × 10${}^{-4}$
	VGGNet-11	0.744	0.712	0.774	0.742	0.727	0.487	5.529 × 10${}^{-8}$
	VGGNet-16	0.739	0.7	0.774	0.739	0.719	0.476	4.612 × 10${}^{-16}$
	AlexNet	0.699	0.659	0.737	0.696	0.677	0.397	3.519 × 10${}^{-2}$
	SqueezeNet1_0	0.719	0.665	0.769	0.724	0.693	0.436	3.130 × 10${}^{-5}$
	SqueezeNet1_1	0.685	0.729	0.645	0.653	0.689	0.375	6.687 × 10${}^{-3}$
Mean subtracted images	DenseNet-121	0.73	0.712	0.747	0.72	0.716	0.459	3.545 × 10${}^{-2}$
	Resnet-18	0.713	0.682	0.742	0.707	0.695	0.425	1.953 × 10${}^{-2}$
	ResNet 34	0.713	0.635	0.785	0.73	0.679	0.426	6.846 × 10${}^{-5}$
	VGGNet-11	0.685	0.735	0.64	0.651	0.691	0.376	5.984 × 10${}^{-4}$
	VGGNet-16	0.725	0.724	0.726	0.707	0.715	0.449	3.542 × 10${}^{-2}$
	AlexNet	0.683	0.688	0.677	0.661	0.674	0.365	2.806 × 10${}^{-3}$
	SqueezeNet1_0	0.677	0.635	0.715	0.671	0.653	0.352	2.390 × 10${}^{-3}$
	SqueezeNet1_1	0.669	0.612	0.72	0.667	0.638	0.334	1.390 × 10${}^{-3}$

**Table 6:** A comparison on the performance of hybrid models built based on both retinal image and DXA data.

Type of Images	DL Model	Accuracy	Sensitivity	Specificity	Precision	f 1 Score	MCC	*p*-Value
Cropped images	DenseNet-121 + DXA	0.74	0.688	0.793	0.771	0.719	0.492	1.371 × 10${}^{-3}$
	ResNet-18 + DXA	0.756	0.666	0.842	0.802	0.725	0.519	1.255 × 10${}^{-3}$
	ResNet-34 + DXA	0.783	0.747	0.816	0.793	0.767	0.566	1.290 × 10${}^{-3}$
	VGGNet-11 + DXA	0.752	0.691	0.812	0.784	0.729	0.512	1.297 × 10${}^{-3}$
	VGGNet-16 + DXA	0.739	0.675	0.8	0.773	0.71	0.49	1.608 × 10${}^{-3}$
	AlexNet + DXA	0.778	0.698	0.854	0.815	0.751	0.559	1.166 × 10${}^{-3}$
	SqueezeNet1_0 + DXA	0.748	0.653	0.836	0.786	0.713	0.498	3.795 × 10${}^{-3}$
	SqueezeNet1_1 + DXA	0.767	0.736	0.795	0.773	0.753	0.534	1.243 × 10${}^{-3}$
Mean subtracted images	DenseNet-121 + DXA	0.736	0.669	0.8	0.76	0.71	0.475	1.409 × 10${}^{-3}$
	ResNet-18 + DXA	0.734	0.639	0.825	0.775	0.699	0.474	1.355 × 10${}^{-3}$
	ResNet-34 + DXA	0.757	0.755	0.761	0.754	0.75	0.52	1.173 × 10${}^{-3}$
	VGGNet-11 + DXA	0.734	0.707	0.760	0.737	0.715	0.474	1.323 × 10${}^{-3}$
	VGGNet-16 + DXA	0.723	0.702	0.746	0.727	0.705	0.456	1.608 × 10${}^{-3}$
	AlexNet + DXA	0.753	0.658	0.841	0.796	0.718	0.511	1.466 × 10${}^{-3}$
	SqueezeNet1_0 + DXA	0.754	0.673	0.829	0.789	0.725	0.511	1.241 × 10${}^{-3}$
	SqueezeNet1_1 + DXA	0.770	0.732	0.804	0.780	0.754	0.540	1.464 × 10${}^{-3}$

**Table 7:** Details of number of participants and number of images used for each age group.

Class	Age Group (18–39)	Age Group (40 and Above)		
	**Participants**	**Images**	**Participants**	**Images**
CVD	115	440	118	434
Control	210	782	40	140

## Data Availability

Restrictions apply to the availability of these data. Data was obtained from QBB under non-disclosure agreement (NDA).

## Supplementary Materials

The following are available online at https://www.mdpi.com/article/10.3390/s22124310/s1. Supplementary File S1: Summary Statistics of the DXA features. Supplementary File S2: Details for parameter optimization for the models. Supplementary File S3: ROC Curve Analysis.

## Abbreviations

The following abbreviations are used in this manuscript:

ASCVD	Atherosclerotic Cardiovascular Disease
AUC	Area Under the Curve
BMI	Body Mass Index
BMD	Bone Mineral Density
CCTA	Coronary CT Angiography
CT	Computed Tomography
CVD	Cardiovascular disease
DL	Deep Learning
DXA	Dual-energy X-ray Absorptiometry
ECG	Electrocardiogram
ECHO	Echocardiography
ML	Machine Learning
MPI	Myocardial Perfusion Imaging
MRI	Magnetic Resonance Imaging
PCG	Phonocardiogram
ROC	Receiver Operating Characteristics
WHO	World Health Organization
WHR	Waist-to-hip Ratio
