# Supplementary material for: Cardiovascular Disease Diagnosis from DXA Scan and Retinal Images Using Deep Learning
Source: Sensors (Basel). 2022 Jun 7;22(12):4310. doi: 10.3390/s22124310 (PMC9228833; doi:10.3390/s22124310)
Supplement: Supplementary file 1 [file sensors-22-04310-s001.zip › Supplementary File S2.pdf]

sensors

Supplementary File S2

Hyperparameter Tuning

Decision Tree (DT) Classifier was tuned where the max_depth parameter was set to 5. Another hyperparameter which is the min_samples_leaf was set to 50, criterion is set to ‘gini’. Max_features were set to ‘None’, 0.0 was set to the ccp_alpha, ‘None’ was set for the random_state, and ‘random’ value was set to the splitter hyperparameter.

Artificial Neural Network (ANN) Classifier hyperparameter tunning resulted in ‘logistic’ being selected for the activation parameter. (100,1) size was set for the hidden_layer_sizes. For the learning_rate hyperparameter, ‘adaptive’ value was selected, and ‘lbfgs’ value was set to the solver hyperparameter. Alpha value was 0.0001, ‘auto’ value was used for the batch_size, max_iter of 200 was used, ‘True’ value was used for shuffle and ‘None’ value was used for the random_state.

Random Forest (RF) Classifier hyperparameter tuning resulted in selecting 10 for the max depth, 14 for the min_samples_leaf, 12 for the min_samples_split, 200 for the n_estimators and 42 for the random_state. ‘gini’ value was used for the criterion, ‘auto’ was used for the max_features parameter, ‘None’ was used for the n_jobes and max_samples parameters, and 0.0 value was used for ccp_alpha

Logistic Regression (LR) Classifier hyperparameters were tuned where for max_iter hyperparamter, the value 1000 was selected. In addition, ‘liblinear’ value was set for the solver hyperparameter. ‘l2’ was used for the penalty, 1.0 was used for the C parameter, ‘None’ was used for the random_state and n_jobs parameters.

Catboost hyperparameters that were tuned include depth which was set to 4. The learning_rate was also tuned where the value 0.04 was selected. Furthermore, 100 value was set to the iterations hyperparameter. Furthermore, ‘Min’ was used for the nan_mode, ‘Logloss’ was used for the eval_metric, 0.10000000149011612 was used for the bayesian_matrix_reg. For the leaf_estimation_method, the value ‘Newton’ was used. 'GreedyLogSum' value was used for the feature_border_type parameter. 0.800000011920929 value was used for the subsample parameter. 4 was used for the random_seed, 16 was used for the max_leaves parameter, 'Cosine' was used for score_function and 10 was used for the leaf_estimation_iterations parameter.

Extreme Gradient Boosting or as famously known XGBoost Classifier was tuned as well for this study, where the max_depth was set to 5. For the n_estimators hyperparameter, 60 was selected and for the learning_rate 0.1 was selected. 0.5 was used for the base_score, ‘gbtree’ was used for the booster, gamma was set to 0. Random_state was set to 43, ‘gain’ was used for the importance_type, 0 was used for the max_delta_step, and ‘exact’ was used for the tree_method. The one (1) value was used for the min_child_weight, num_parallel_tree, reg_lambda and scale_pos_weight parameters.
