# Supplementary material for: Cardiovascular Disease Diagnosis from DXA Scan and Retinal Images Using Deep Learning
Source: Sensors (Basel). 2022 Jun 7;22(12):4310. doi: 10.3390/s22124310 (PMC9228833; doi:10.3390/s22124310)

sensors

Supplementary File S3

ROC Analysis

The following figure shows the ROC curve and the calculated AUC for all Deep learning models that were tested for the Hybrid model using the cropped images.

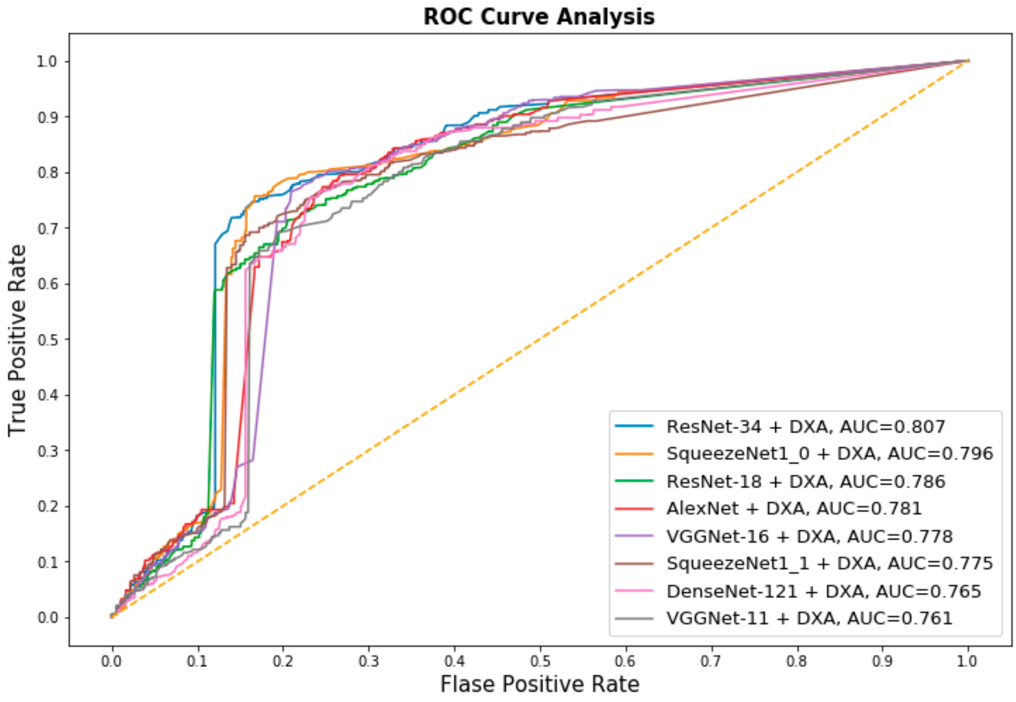

Supplement: Supplementary file 1 [file sensors-22-04310-s001.zip › Supplementary File S3.pdf]
